# Impact of Pneumococcal Vaccination on the Occurrence of Complicated Pneumonia in Children: A Retrospective Analysis

**DOI:** 10.3390/life16050858

**Published:** 2026-05-21

**Authors:** Katarina Milosevic, Jasna Kalanj, Nadja Cukanovic, Luka Zekovic, Vesna Selakovic, Snezana Rsovac

**Affiliations:** 1 Department of Allergology and Immunology, University Children’s Hospital, Tirsova 10, 11000 Belgrade, Serbia; 2 Faculty of Medicine, University of Belgrade, Dr Subotica 8, 11000 Belgrade, Serbia; 3 Department of Pediatric and Neonatal Intensive Care, University Children’s Hospital, Tirsova 10, 11000 Belgrade, Serbia

**Keywords:** complicated pneumonia, empyema, pneumococcal vaccine, lung abscess, children, fibrinolytic therapy

## Abstract

Complicated community-acquired pneumonia (cCAP) remains a major cause of morbidity in children. Although pneumococcal conjugate vaccines (PCVs) have reduced invasive disease, severe complications such as empyema and lung abscess persist. A retrospective analysis was conducted on 69 children treated at the University Children’s Hospital Belgrade between 2019 and 2024. Data included demographic characteristics, pneumococcal vaccination status, and radiologically confirmed complications. Patients were classified by residence and vaccination status. Statistical analysis included chi-square (χ^2^) tests, odds ratios (ORs) with 95% confidence intervals, and multivariable logistic regression. Pleuropneumonia and pleural effusion were the most frequent complications, while empyema and lung abscess were the most severe. Both occurred significantly more often in unvaccinated children (*p* = 0.0054 and *p* = 0.0027). Multivariable analysis confirmed vaccination as an independent protective factor against empyema (adjusted OR = 0.19, 95% CI 0.06–0.61). No significant regional differences were observed after accounting for vaccination status. Vaccination showed a strong protective effect against empyema and lung abscess (OR = 0.24 and 0.04, respectively). Unvaccinated children had significantly longer hospital stays, indicating a more severe clinical course. Prolonged hospitalization was associated with intensified antibiotic therapy, reflecting underlying disease severity. Lack of pneumococcal vaccination is strongly associated with severe complications in children with cCAP. Maintaining high PCV coverage remains essential, alongside early recognition and timely management of pleural disease.

## 1. Introduction

Complicated community-acquired pneumonia (cCAP) remains one of the leading causes of pediatric hospitalization and morbidity worldwide, representing a significant burden on healthcare systems and families alike. Despite the widespread implementation of pneumococcal conjugate vaccines (PCVs), severe pleuropulmonary complications—including empyema, lung abscess, pleural effusion, and necrotizing pneumonia—continue to impose substantial clinical, economic, and psychosocial burdens [1,2]. These complications often necessitate prolonged hospitalization, intensive supportive care, invasive procedures such as thoracentesis or chest tube placement, and occasionally surgical intervention, all of which contribute to increased healthcare costs and strain on hospital resources. Intrapleural fibrinolytic therapy, most commonly using tissue plasminogen activator (tPA), represents a key therapeutic approach in the management of empyema by facilitating the breakdown of fibrin septations within the pleural space and improving fluid drainage [3].

Recent epidemiological studies indicate that the persistence of complicated disease may result from multiple interconnected factors. Shifts in pneumococcal serotype distribution, particularly the emergence of non-vaccine serotypes following widespread PCV implementation, have been implicated in sustaining the incidence of severe disease [4,5]. These serotype shifts can result in incomplete herd immunity and may alter patterns of disease severity. Additionally, residual non-vaccine serotypes may possess enhanced virulence or a higher propensity for causing pleural and parenchymal complications, further contributing to persistent rates of empyema and lung abscess. Post-pandemic disruptions in healthcare access, including delays in routine pediatric visits, vaccination schedules, and early diagnosis, have further complicated the epidemiological landscape, potentially allowing infections to progress to more severe forms before clinical intervention [4,5].

Among the spectrum of complicated pneumonia, empyema and pleural effusion remain among the most frequent and resource-intensive complications. Large multicenter analyses over the past five years have consistently reported that children with pleural involvement experience prolonged hospital stays, an increased need for invasive diagnostic and therapeutic procedures, and a higher likelihood of long-term pulmonary sequelae, including restrictive lung disease and impaired exercise tolerance [6,7]. These outcomes underscore the importance of early detection and timely management of pleural complications to prevent progression and reduce morbidity.

Although pneumococcal vaccination has significantly reduced the incidence of invasive pneumococcal disease, bacteremia, and overall pediatric mortality, its impact on complicated pneumococcal pneumonia varies across regions and populations [2,6]. Contemporary vaccine effectiveness studies indicate that PCVs provide sustained protection against empyema and lung abscess caused by vaccine serotypes; however, challenges remain due to serotype replacement, suboptimal vaccination coverage, and variability in host immune responses [4,8]. These findings highlight that vaccination alone, while crucial, is not sufficient to eliminate the burden of severe pulmonary complications and must be complemented by timely clinical recognition and evidence-based management strategies. In Serbia, there is a paucity of systematic data evaluating the interplay between pneumococcal vaccination status, regional residence, and the spectrum of pulmonary complications in children with cCAP. Previous institutional studies from our center have demonstrated that intrapleural fibrinolytic therapy significantly improves outcomes in children with empyema and complicated pneumonia, reducing the need for surgical intervention and accelerating clinical recovery [9]. Despite these advances, the specific impact of vaccination on the occurrence, severity, and regional distribution of these complications has not been fully characterized. Addressing this knowledge gap is essential for optimizing both preventive and therapeutic strategies, guiding national immunization policies, and informing resource allocation within pediatric healthcare systems.

Furthermore, understanding the epidemiological patterns of cCAP and its complications in the context of vaccination coverage can inform public health initiatives aimed at increasing PCV uptake, identifying at-risk populations, and developing targeted interventions to reduce morbidity. The combination of vaccination, early recognition of clinical deterioration, timely imaging, and prompt interventional management constitutes a comprehensive approach to mitigating the burden of complicated pneumonia in pediatric populations.

## 2. Materials and Methods

A retrospective study was conducted including 69 children diagnosed with complicated community-acquired pneumonia who were treated at the University Children’s Hospital Belgrade between January 2019 and December 2024. The diagnosis of pneumonia was established based on a combination of clinical presentation and radiographic findings, in accordance with current pediatric and infectious disease guidelines. Clinical criteria included the presence of fever, cough, tachypnea, respiratory distress, and auscultatory findings such as crackles or decreased breath sounds, while radiographic confirmation was obtained using chest X-ray and, when clinically indicated, additional imaging modalities such as lung ultrasound or computed tomography (CT).

Complicated pneumonia was defined by the presence of one or more radiologically confirmed conditions, including pleural effusion, empyema, lung abscess, pneumothorax, pyothorax, pleuropneumonia, atelectasis, or emphysema. These complications represent different stages and manifestations of disease progression, ranging from fluid accumulation in the pleural space to necrotizing and suppurative processes within the lung parenchyma. The classification of complications was based on standardized radiological criteria and validated by experienced pediatric radiologists, ensuring consistency and diagnostic reliability throughout the study period.

For the purposes of this study, “pneumonia” was defined as cases of radiologically confirmed pneumonia without local suppurative complications but requiring parenteral administration of reserve antibiotics due to clinical severity or inadequate response to first-line therapy [10,11].

Pleuropneumonia was defined as pneumonia associated with inflammatory involvement of the pleura, with or without pleural effusion [12].

Empyema was defined as the presence of purulent pleural fluid confirmed by imaging and/or pleural fluid analysis. In contrast, the term “pyothorax” was used to describe the presence of pus within the pleural cavity occurring as a complication of invasive pleural procedures, such as drainage or thoracic interventions [13,14].

Patients were classified according to pneumococcal vaccination status as vaccinated or unvaccinated, based on documented immunization records obtained from medical charts and vaccination registries when available. Only children who had received the complete age-appropriate pneumococcal vaccination schedule were considered vaccinated. In this cohort, all vaccinated patients born before 2018 received the 7-valent pneumococcal conjugate vaccine (PCV7). Pneumococcal vaccination was not included in the mandatory immunization schedule in Serbia prior to 2018, when pneumococcal conjugate vaccines (PCV10 and later PCV13) were introduced into routine childhood immunization in accordance with national guidelines issued by the Institute of Public Health of Serbia “Dr Milan Jovanović Batut”. Information regarding the timing of vaccination relative to disease onset was also reviewed when available, although it was not used as a separate analytical variable.

In addition to vaccination status, patients were stratified according to region of residence into three categories: Belgrade, other regions of Serbia, and outside Serbia. This classification was included to explore potential differences in disease presentation, access to healthcare, and referral patterns between urban and non-urban populations, as well as between domestic and international patients. Demographic variables collected for all participants included age and sex, allowing for descriptive analysis of the study population and identification of potential demographic influences on disease severity.

Each complication was analyzed individually, acknowledging that a single patient could present with multiple simultaneous complications. This approach allowed for a more detailed characterization of the clinical spectrum of complicated pneumonia and avoided underestimation of disease burden. The presence of multiple complications in the same patient was recorded and included in the analysis, reflecting the complex and often progressive nature of severe pulmonary infections in children.

All patients were managed according to contemporary clinical protocols and international guidelines for the treatment of pediatric pneumonia and its complications. Empirical antibiotic therapy in accordance with institutional protocols and international pediatric guidelines [15]. In the absence of β-lactam allergy, third-generation cephalosporins, such as ceftriaxone or cefotaxime, were used as first-line treatment. Cefotaxime was preferred in younger infants due to its favorable safety profile, whereas ceftriaxone was more commonly used in older children. Antibiotic therapy was subsequently adjusted based on clinical response and microbiological findings.

Standard treatment included early initiation of empiric antibiotic therapy, supportive care, and close clinical monitoring. In cases with significant pleural involvement, interventions such as pleural drainage and intrapleural fibrinolytic therapy were performed when indicated. Although treatment modalities were not the primary focus of this analysis, their consistent application across patients contributed to the overall comparability of outcomes. Data on intrapleural fibrinolytic therapy was collected, including whether fibrinolysis was performed during hospitalization. Fibrinolysis was defined as the administration of intrapleural fibrinolytic agents (e.g., tissue plasminogen activator, streptokinase, dornase alfa) as part of the treatment of pleural infection. Data on pleural drainage, length of hospital stay, and pre-hospital antibiotic use were also collected for all patients.

Statistical analysis was performed using SPSSAU v24.0 (SPSSAU Cloud Statistical Analysis Platform, 4th Generation Statistical Analysis Software; https://spssau.net (accessed on 18 May 2026)). Continuous variables were expressed as median (interquartile range, IQR), while categorical variables were presented as frequencies and percentages. Comparisons between groups were performed using the chi-square test or Fisher’s exact test, as appropriate. Binary logistic regression analysis was used to calculate odds ratios (OR) with 95% confidence intervals (CI). A *p*-value < 0.05 was considered statistically significant. The Mann–Whitney U test was used for comparisons between groups when data were not normally distributed.

Multivariate logistic regression analysis was performed to evaluate the independent association between pneumococcal vaccination status and pulmonary complications (empyema and lung abscess), adjusting for potential confounders including age and sex. Results are presented as odds ratios (OR) with 95% confidence intervals (CI). Model fit was assessed using the likelihood ratio test and Hosmer–Lemeshow goodness-of-fit test.

Odds ratios (ORs) with 95% confidence intervals were calculated to evaluate the strength of association between vaccination status and the occurrence of individual complications. This measure was chosen to provide a clinically meaningful estimate of relative risk in this retrospective cohort. All statistical tests were two-tailed, and a *p*-value < 0.05 was considered statistically significant.

To ensure data accuracy and consistency, all extracted data were independently reviewed and cross-checked by multiple investigators involved in the study. Any discrepancies were resolved through consensus. Although the retrospective design inherently carries limitations, including potential selection bias and incomplete data capture, careful methodological planning and standardized definitions were used to minimize these effects and enhance the validity of the findings.

## 3. Results

The study included 69 children with cCAP, with a slight male predominance (40 male, 29 female) (Table 1). All patients were managed in accordance with current international guidelines and contemporary pediatric practice, including early initiation of antibiotic therapy, timely pleural drainage, and selective use of intrapleural fibrinolytic therapy. The most frequent complications were pleuropneumonia and pleural effusion, followed by empyema and lung abscess (Table 1).

Microbiological pathogen was isolated in 17 (24.6%) patients. Among these, antibiotic multiresistance was detected in 8 (47.1%) cases.

Intrapleural fibrinolytic therapy was performed in 22 out of 69 patients (31.9%). It was more frequently applied in vaccinated patients (36.1%) compared to unvaccinated patients (27.3%); however, this difference was not statistically significant (*p* > 0.05). Furthermore, binary logistic regression analysis confirmed that vaccination status was not a significant predictor of fibrinolysis (OR = 1.51, *p* = 0.432).

Pleural drainage was performed in 48 out of 69 patients (69.6%). It was more frequently required in unvaccinated patients (75.8%) compared to vaccinated patients (63.9%); however, this difference was not statistically significant (*p* = 0.284).

**Figure 1:**
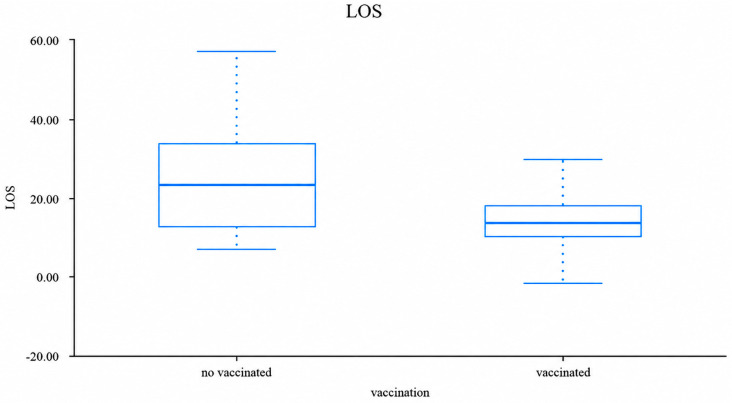
Length of hospital stay according to vaccination status. Boxplot showing the distribution of length of hospital stay in vaccinated and unvaccinated patients. Unvaccinated children had significantly longer hospitalization duration (*p* < 0.01).

The median length of hospital stay (LOS) was significantly longer in unvaccinated patients compared to vaccinated patients (23.0 days [IQR 12.5–33.5] vs. 13.5 days [IQR 10.0–17.8], *p* < 0.01) (Figure 1).

Patients receiving reserve antibiotics had significantly longer hospital stays compared to those who did not (median 17 vs. 9.5 days, *p* = 0.016). Similarly, combination antibiotic therapy was associated with prolonged hospitalization (17 vs. 10 days, *p* = 0.038). Escalation of antibiotic therapy showed the strongest association with length of stay, with significantly longer hospitalization in patients requiring escalation (23 vs. 14 days, *p* = 0.009).

Pre-hospital antibiotic therapy was recorded in 39 out of 69 patients (56.5%). Empyema was more frequently observed among patients who had received antibiotics prior to admission (68.0%) compared to those who had not (32.0%); however, this difference was not statistically significant (*p* > 0.05).

No significant correlation was observed between time to initiation of antibiotic therapy and length of hospital stay (Spearman r = 0.11, *p* = 0.381).

Baseline characteristics differed between groups only in terms of sex distribution, with a higher proportion of males in the unvaccinated group (72.7% vs. 44.4%, *p* = 0.017). No significant differences were observed in age (*p* > 0.05) or time to antibiotic initiation (median 5.0 vs. 5.0 days, *p* = 0.894) (Table 2).

When complications were compared according to vaccination status, empyema and lung abscess were found to be significantly more frequent among unvaccinated children (*p* = 0.006 and *p* = 0.0027, respectively) (Table 3). Empyema remained significantly more frequent among unvaccinated children (54.5% vs. 22.2%), with vaccination associated with lower odds of empyema (OR = 0.24, 95% CI 0.08–0.66, *p* = 0.006. Pleuropneumonia was more frequently observed among vaccinated patients (58.3%) compared to unvaccinated patients (42.4%); however, this difference was not statistically significant (OR = 1.90, 95% CI 0.70–5.10, *p* = 0.187). Other complications, including pneumonia, pleuropneumonia, atelectasis, pneumothorax, pleural effusion, pyothorax, and emphysema, showed no statistically significant difference between vaccinated and unvaccinated groups.

**Figure 2:**
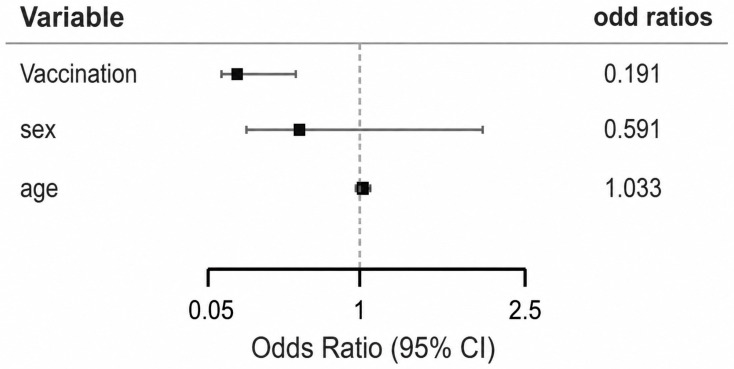
Forest plot of multivariable logistic regression analysis showing odds ratios (OR) with 95% confidence intervals (CI) for predictors of empyema. Black squares represent odds ratios, horizontal lines indicate 95% confidence intervals. Vaccination was independently associated with significantly lower odds of empyema, whereas age and sex were not significant predictors.

Multivariate logistic regression analysis demonstrated that pneumococcal vaccination remained an independent protective factor against empyema after adjustment for age and sex (OR = 0.19, 95% CI 0.06–0.61, *p* = 0.005). Neither age nor sex were significantly associated with empyema (Figure 2). The overall model was statistically significant (χ^2^ = 9.24, *p* = 0.026) and demonstrated good goodness-of-fit (Hosmer–Lemeshow *p* = 0.417). Due to the low number of lung abscess cases, multivariate estimates were unstable and should be interpreted with caution.

Descriptive analysis by region showed that the majority of patients originated from Belgrade (55.1%), followed by other regions of Serbia (37.7%) and outside Serbia (7.2%).

Vaccination coverage varied across regions, with the highest proportion observed in Belgrade (55.3%) compared to other regions of Serbia (42.3%) and outside Serbia (50.0%). Empyema was slightly more frequent in patients from other regions of Serbia (38.5%) compared to Belgrade (28.9%), while lung abscess was infrequent across all regions and showed no clear regional pattern (Table 4).

**Figure 3:**
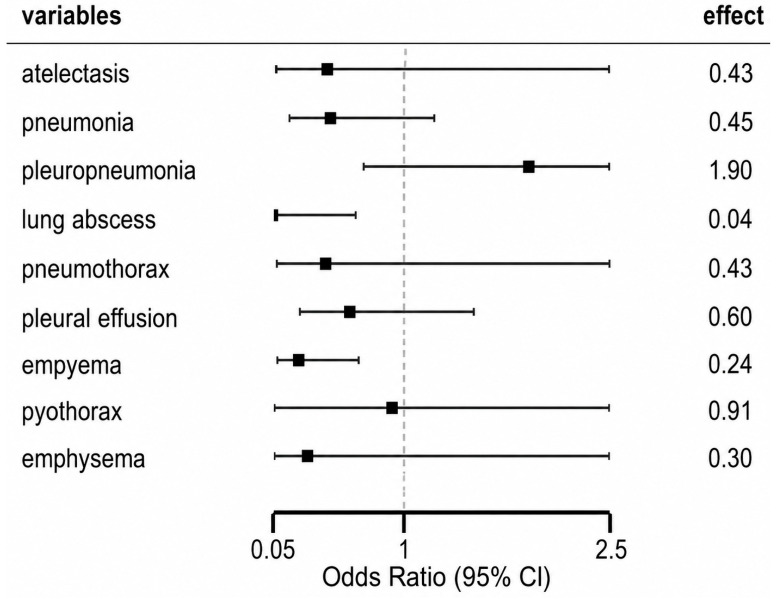
Forest plot: Forest plot showing odds ratios (ORs) with 95% confidence intervals (CIs) for pulmonary complications according to pneumococcal vaccination status. Black squares represents odds ratios, horizontal lines indicate 95% confidence intervals, and dashed vertical line represents the null value (OR = 1). Vaccination was significantly associated with lower odds of empyema and lung abscess, while no statistically significant differences were observed for other complications.

Odds ratio analysis showed an increased risk of empyema and lung abscess among unvaccinated patients, confirming the association between lack of vaccination and more severe disease manifestations (Figure 3).

## 4. Discussion

Our findings demonstrate a strong and clinically meaningful association between the lack of pneumococcal vaccination and the increased occurrence of empyema and lung abscess in children with complicated community-acquired pneumonia. These results are fully consistent with contemporary international evidence confirming the protective role of pneumococcal conjugate vaccines (PCVs) against invasive and complicated pneumococcal disease [1,4,6]. The magnitude of this association observed in our cohort further emphasizes that vaccination does not merely reduce disease incidence but also significantly modifies disease severity and clinical trajectory. Although the widespread implementation of PCVs has substantially reduced the overall burden of severe pneumococcal infections, complicated pneumonia remains a significant clinical challenge, particularly in regions with suboptimal vaccination coverage, delayed healthcare access, and ongoing serotype replacement [2,8].

In addition to univariate analyses, multivariable logistic regression was performed to further evaluate the independent effect of vaccination status on the occurrence of empyema. After adjustment for age and sex, vaccination remained a significant protective factor, reducing the odds of empyema by approximately 75% (OR = 0.243, 95% CI: 0.080–0.733, *p* = 0.012). This finding confirms that the observed association is not confounded by basic demographic variables and strengthens the conclusion that vaccination independently modifies disease severity, consistent with previous studies demonstrating the effectiveness of pneumococcal conjugate vaccines in reducing severe and invasive disease manifestations [6,10,11].

A separate model for lung abscess could not be reliably interpreted due to complete separation, as no cases were observed in the vaccinated group, resulting in unstable estimates. The absence of lung abscess cases in the vaccinated group resulted in complete separation, limiting the reliability of regression estimates and suggesting that the strength of association may be overestimated. Nevertheless, this pattern further supports a strong protective effect of vaccination against the most severe suppurative complications, in line with epidemiological data indicating a marked reduction in advanced pleuropulmonary complications following widespread PCV implementation [4,5].

From a pathophysiological perspective, the protective effect of vaccination may be explained by the reduction in nasopharyngeal colonization with vaccine serotypes, leading to decreased bacterial load and reduced likelihood of invasive spread into the pleural space and lung parenchyma. In unvaccinated children, higher bacterial virulence and delayed immune response may facilitate the progression from uncomplicated pneumonia to necrotizing disease, empyema, or abscess formation. This biological plausibility further strengthens the causal interpretation of our findings.

Despite the proven effectiveness of vaccination, our cohort still demonstrated a considerable burden of pleural effusion and pleuropneumonia even among vaccinated children. This observation aligns with recent multicenter studies showing that vaccination, while highly effective against vaccine-covered serotypes, does not completely eliminate complicated pneumonia [5,7]. One possible explanation lies in the phenomenon of serotype replacement; whereby non-vaccine pneumococcal serotypes increasingly occupy the ecological niche left by vaccine-targeted strains. These emerging serotypes may differ in virulence, transmissibility, and propensity to cause pleural disease.

In addition to pneumococcal factors, non-pneumococcal bacterial pathogens such as Staphylococcus aureus and Streptococcus pyogenes have become increasingly important contributors to severe pulmonary infections in the post-PCV era [7,8]. These pathogens are often associated with more aggressive clinical courses, including rapid progression to empyema or necrotizing pneumonia. Furthermore, viral–bacterial co-infections have been identified as important modulators of disease severity, particularly following the COVID-19 pandemic, during which alterations in viral circulation patterns and immune responses may have influenced susceptibility to secondary bacterial infections [5].

The significantly higher incidence of empyema and lung abscess among unvaccinated children observed in our study further supports the critical importance of pneumococcal vaccination as a primary preventive strategy. Empyema represents one of the most severe and resource-intensive complications of pediatric pneumonia, frequently requiring invasive procedures, prolonged hospitalization, and multidisciplinary care [6]. Beyond immediate clinical burden, empyema may also be associated with long-term sequelae, including restrictive lung disease and impaired pulmonary function, particularly in cases with delayed or inadequate treatment.

Our regional analysis demonstrated that once vaccination status was considered, geographic residence did not independently influence the distribution of severe complications. Regional comparisons should be interpreted with caution, as referral bias may have influenced the distribution of more severe cases, particularly among patients transferred from outside Belgrade. This finding underscores that vaccination status, rather than regional factors alone, represents the dominant determinant of disease severity in our population. Nevertheless, regional differences in healthcare infrastructure, referral pathways, and timing of presentation may still play a secondary role, particularly in influencing disease progression prior to hospital admission.

Management followed current international guidelines and contemporary pediatric practice, with early antibiotic administration, prompt pleural drainage, and selective intrapleural fibrinolytic therapy [2,6]. Multiple pediatric studies over the last five years have confirmed that intrapleural alteplase (tPA) is both safe and effective, significantly reducing the need for surgical intervention and shortening hospital stay in children with empyema [16,17]. The mechanism of fibrinolytic therapy involves the breakdown of fibrin septations within the pleural space, facilitating drainage and improving lung re-expansion. In our cohort, the use of fibrinolysis did not differ significantly between vaccinated and unvaccinated patients, indicating that the decision to initiate fibrinolytic therapy was primarily driven by disease severity rather than vaccination status. Therefore, fibrinolysis should be interpreted as a marker of disease severity rather than a determinant of clinical outcomes in this cohort. The lack of association between fibrinolysis and vaccination status likely reflects confounding by indication, as fibrinolytic therapy is primarily administered in patients with more severe pleural disease. This finding further supports the concept that fibrinolysis represents a therapeutic response to advanced pleural disease rather than a factor influenced by preventive measures such as vaccination.

Similarly, pleural drainage was frequently required, particularly in patients with empyema, but did not differ significantly between vaccination groups, suggesting that the need for invasive intervention is primarily determined by disease severity rather than vaccination status alone. These findings collectively suggest that vaccination influences not only the occurrence of complications but also the overall clinical course and healthcare resource utilization.

The significantly longer hospital stay observed in unvaccinated patients further highlights the clinical impact of vaccination, suggesting that pneumococcal immunization not only reduces the risk of severe complications but also shortens disease course and hospitalization duration. Although pre-hospital antibiotic use was more frequent among patients who developed empyema, this association was not statistically significant. This finding likely reflects delayed presentation, disease severity at initial evaluation, or suboptimal early treatment rather than causal effect of antibiotic therapy itself [18]. The observed higher frequency of empyema among patients who received pre-hospital antibiotics likely reflects confounding by disease severity and delayed presentation, rather than a direct effect of antibiotic therapy.

In our cohort, prolonged hospital stay was strongly associated with the need for intensified antibiotic management, including the use of reserve agents, combination therapy, and particularly escalation of initial empirical treatment, reflecting underlying disease severity rather than a causal effect of antibiotic therapy itself. This observation is consistent with data from European pediatric centers, where treatment escalation is typically required in patients with more advanced or non-responsive disease and is associated with more complicated clinical courses and prolonged hospitalization [19]. In contrast, we did not observe a significant correlation between time to antibiotic initiation and length of hospital stay, suggesting that variability in treatment timing did not independently influence disease course. These findings support the concept that disease progression is primarily driven by factors such as pathogen virulence, host response, and stage at presentation, rather than antibiotic timing alone.

These data are fully supported by our previously published MAR study, which demonstrated that fibrinolytic therapy was associated with faster radiological resolution, improved clinical recovery, and reduced rates of surgical referral [9]. The present findings further reinforce the clinical value of fibrinolysis as a cornerstone of modern empyema management and highlight the importance of early identification of patients who may benefit from this intervention.

The persistence of pneumothorax and pleural effusion across both vaccinated and unvaccinated groups, without statistically significant differences, reflects the multifactorial nature of complicated pneumonia. These complications may arise from disease progression, delayed presentation, mechanical ventilation, or underlying lung pathology, rather than direct pneumococcal virulence alone [6,7]. Additionally, host-related factors such as age, immune status, and genetic susceptibility may influence individual risk of developing specific complications. This highlights the importance of early diagnosis, timely imaging, and structured treatment pathways in preventing the escalation of disease severity.

From a public health standpoint, the results of this study carry important implications. The clear association between non-vaccination and severe complications strongly reinforces the necessity of maintaining high PCV coverage at the national level. Recent epidemiological analyses emphasize that even temporary declines in vaccine uptake can result in a measurable increase in severe pneumococcal disease and empyema incidence within a relatively short period [4,5]. This is particularly relevant in the context of vaccine hesitancy and disruptions to routine immunization programs observed during the COVID-19 pandemic.

In this context, targeted vaccination campaigns, systematic verification of immunization status at hospital admission, and implementation of catch-up vaccination programs remain essential components of pediatric infection control strategies. In addition, continuous surveillance of pneumococcal serotypes and antimicrobial resistance patterns is crucial for guiding future vaccine development and optimizing empirical treatment protocols.

Beyond prevention, our findings also highlight the importance of regional healthcare organization and timely access to specialized care. Early referral to tertiary pediatric centers with expertise in interventional pulmonology, pediatric surgery, and fibrinolytic therapy significantly influences outcomes in children with complicated pneumonia [6,9,10]. Delayed transfer and late initiation of drainage or fibrinolysis have been repeatedly associated with prolonged hospitalization, increased healthcare costs, and a higher risk of chronic pleural disease. Strengthening referral networks and ensuring standardized treatment protocols across healthcare levels should therefore be considered a priority.

Several limitations of the present study should be acknowledged. The retrospective design inherently carries a risk of selection bias, incomplete data capture, and limited control over confounding variables. As multiple complications could occur in the same patient, the analyzed outcomes were not fully independent, which may have influenced the observed associations. The relatively modest sample size resulted in wide confidence intervals for some odds ratio estimates, which may limit the precision of risk assessment. The relatively small sample size may have limited statistical power for detecting differences in less frequent complications and contributed to wide confidence intervals. Despite these limitations, the consistency of findings across multiple analytical approaches strengthens the robustness of the observed associations.

Nevertheless, the strength of our study lies in the integration of detailed vaccination data, regional stratification, and comprehensive complication profiling within a clearly defined tertiary-care cohort. The consistency of our findings with international literature further supports their validity and clinical relevance, providing important regional evidence for the protective role of pneumococcal vaccination in preventing severe complications of pediatric pneumonia.

## 5. Conclusions

This study provides strong regional evidence that pneumococcal vaccination significantly reduces the occurrence of the most severe pulmonary complications—particularly empyema and lung abscess—in children with complicated community-acquired pneumonia. These findings reinforce the central role of pneumococcal conjugate vaccines not only in preventing invasive disease but also in mitigating the progression toward advanced and life-threatening pulmonary manifestations. Importantly, our results highlight that vaccination contributes not only to a reduction in disease incidence but also to a modification of clinical severity, with vaccinated children demonstrating a markedly lower risk of developing necrotizing and suppurative complications. This has direct implications for clinical outcomes, including shorter hospital stays, reduced need for invasive procedures, and lower risk of long-term respiratory sequelae. Combined with timely pleural drainage and selective intrapleural fibrinolytic therapy, high vaccination coverage remains the most effective strategy for reducing both morbidity and healthcare burden associated with pediatric complicated pneumonia. Early recognition of disease progression, appropriate imaging, and prompt initiation of evidence-based therapeutic interventions are essential components of optimal patient management and further contribute to improved outcomes.

From a broader public health perspective, these findings underscore the critical importance of maintaining and continuously improving pneumococcal vaccination coverage within national immunization programs. Efforts to address vaccine hesitancy, ensure equitable access to immunization, and implement catch-up vaccination strategies are essential to preventing severe disease forms.

Future research should focus on larger prospective cohorts, incorporation of detailed microbiological and serotype-specific data, and evaluation of newer-generation pneumococcal vaccines to further clarify their impact on complicated pneumonia. Continuous epidemiological surveillance and integration of clinical and public health strategies will be crucial for sustaining progress in the prevention and management of pediatric respiratory infections.

## Figures and Tables

**Table 1:** Demographic characteristics, vaccination status, and complications of study participants. Complication categories are not mutually exclusive; individual patients may have had multiple complications.

Variable	*n* (%) or Mean (IQR)
**Number of participants**	69
**Age (years)**	4.5 (2.5–10.6)
**Sex**	
Male	40 (58.0%)
Female	29 (42.0%)
**Vaccination status**	
Vaccinated	36 (52.2%)
Unvaccinated	33 (47.8%)
**Microbiological findings**	
Pathogen isolated	17 (24.6%)
No pathogen isolated	52 (75.4%)
Antibiotic multiresistance	8 (47.1%)
**Complications**	
Pneumonia	39 (56.5%)
Pleuropneumonia	35 (50.7%)
Pleural effusion	29 (42.0%)
Empyema	26 (37.7%)
Lung abscess	9 (13.0%)
Pneumothorax	6 (8.7%)
Atelectasis	6 (8.7%)
Pyothorax	2 (2.9%)
Emphysema	1 (1.4%)

**Table 2:** Baseline characteristics of the study population by vaccination status.

Variable	Vaccinated n (%) or Median (IQR) (n = 36) n (52.2%)	Vaccinated n (%) or Median (IQR) (n = 33) n (47.8%)	*p*-Value
Age	27.5 (15.3–41.3)	31.0 (18.0–41.5)	0.513
Sex			**0.017**
Male	16 (44%)	24 (72.7%)	
Female	20 (55.6%)	9 (27.3%)	
Duration of symptoms before first dose of antibiotic	5.0 (4.0–8.0)	5.0 (4.0–7.5)	0.894

**Table 3:** Comparison of complications by vaccination status. Complication categories are not mutually exclusive; individual patients may have had multiple complications. OR < 1 indicates lower odds among vaccinated children.

Complication	Vaccinated n (%) (n = 36) n (52.2%)	Unvaccinated n (%) (n = 33) n (47.8%)	OR	95% CI	χ^2^ Test (*p*-Value)
Pneumonia	17 (47.2%)	22 (66.7%)	0.45	0.17–1.20	0.104
Pleuropneumonia	21 (58.3%)	14 (42.4%)	1.90	0.70–5.10	0.187
Pleural effusion	13 (36.1%)	16 (48.5%)	0.60	0.24–1.50	0.298
Empyema	8 (22.2%)	18 (54.5%)	**0.24**	**0.08–0.66**	0.006
Lung abscess	0 (0.0%)	9 (27.3%)	0.04 *	**0.002–0.64**	<0.001
Pneumothorax	2 (5.6%)	4 (12.1%)	0.43	0.07–2.50	0.334
Atelectasis	2 (5.6%)	4 (12.1%)	0.43	0.07–2.50	0.334
Pyothorax	1 (2.8%)	1 (3.0%)	0.91	0.05–15.23	0.950
Emphysema	0 (0.0%)	1 (3.0%)	0.30	0.01–7.54	0.293

* Odds ratios for zero cell counts were estimated using the Haldane–Anscombe correction.

**Table 4:** Values are presented as number (N) and percentage within each region. Vaccination status and complication frequencies were calculated relative to the total number of patients in each regional subgroup.

Region	N	Vaccinated (%)	Empyema (%)	Lung Abscess (%)
Belgrade	38	55.3%	28.9%	13.2%
Other Serbia	26	42.3%	38.5%	11.5%
Outside Serbia	5	60.0%	20.0%	0.0%

## Data Availability

Dataset available on request from the authors.

## Abbreviations

The following abbreviations are used in this manuscript:

OR	Odds ratio
CI	Confidence interval
SD	Standard deviation
LOS	Length of Stay
